# History of Recurrent Implantation Failure is Associated With the Incidence of Adverse Perinatal Outcomes in Singleton Live Births Following Frozen-Thawed Embryo Transfer Cycles

**DOI:** 10.3389/fendo.2021.774646

**Published:** 2022-02-08

**Authors:** Na Li, Yichun Guan, Junjie Liu, Bingnan Ren, Yulin Du, Kexin Wang, Yongjie Zhang, Hua Lou

**Affiliations:** ^1^ Reproductive Center, The Third Affiliated Hospital of Zhengzhou University, Zhengzhou, China; ^2^ Henan Human Sperm Bank, The Third Affiliated Hospital of Zhengzhou University, Zhengzhou, China

**Keywords:** recurrent implantation failure, frozen-thawed embryo transfer, premature rupture of the membranes, perinatal outcomes, intrauterine procedures

## Abstract

**Objective:**

To investigate whether patients with a history of recurrent implantation failure (RIF) are associated with adverse perinatal outcomes in singleton live births following frozen-thawed embryo transfer (FET) cycles.

**Design:**

Retrospective cohort study.

**Methods:**

This study analyzed the obstetric and neonatal outcomes of patients with and without a history of RIF who underwent FET cycles in a single reproductive center between January 2017 and October 2020. A total of 1,100 women with singleton live births beyond 28 weeks of gestation were included. The primary outcome measures were perinatal outcomes, especially gestational age, birthweight, preterm birth (PTB), large for gestational age (LGA), small for gestational age (SGA), congenital malformation rates, and premature rupture of the membranes (PROM). Multiple logistic regression was used to establish relationships between RIF and adverse perinatal outcomes after adjusting for relevant baseline demographics and cycle characteristics.

**Result(s):**

The RIF group showed a preferred transfer of two embryos and cleavage embryos compared with the control group (*P <*0.05). Regarding perinatal outcomes in singleton deliveries, women with RIF had increased rates of LBW (adjusted odds ratio [aOR] 2.027; 95% confidence interval [CI], 1.025–4.009), PTB (aOR 1.785; 95% CI, 1.050–3.036), and PROM (aOR 2.259; 95% CI, 1.142–4.467). The incidence of congenital malformations was similar between the two groups (4.1% vs. 2.4%; *P* = 0.759). Furthermore, multiple intrauterine procedures were associated with a statistically significant increased risk of PROM in RIF patients (aOR 1.537; 95% CI, 1.105–2.137).

**Conclusions:**

Women with a history of RIF were associated with an increased risk of LBW, PTB, and PROM in singleton live births after FET cycles. In addition, multiple intrauterine procedures were independent risk factors for PROM.

## Introduction

Assisted reproductive technology (ART) has improved substantially over the past four decades and has brought hope to thousands of infertile families (1). Nevertheless, many couples still fail to become pregnant for unexplained reasons, even after multiple attempts at embryo transfer, which undoubtedly places tremendous mental pressure and economic burden on the patients and their families. This is often described as recurrent implantation failure (RIF); so far, there is no universally accepted consensus on the definition of RIF. Coughlan et al. (2) recently proposed that “women under the age of 40 years who experienced at least three fresh or frozen-thawed embryo transfer (FET) cycles and cumulatively transferred at least four good-quality embryos without getting clinical pregnancy” were considered to be RIF.

Embryo implantation is a complex process that depends on embryo quality, endometrial receptivity, and the communication between embryos and the endometrium. Over the last two decades, the management of RIF has included preimplantation genetic testing for aneuploidy in order to select only euploid embryos for transfer, or extended culture leading to embryo transfer at the blastocyst-stage, or the practice of hysteroscopy to eliminate an abnormal uterine cavity (3). Recently, a number of procedures, namely, the administration of endometrial scraping and perfusion of granulocyte colony-stimulating factor (G-CSF) before embryo transfer have been developed to improve endometrial receptivity and decrease the incidence of implantation failure in *in vitro* fertilization (IVF) cycles (4–6).

Previous studies have addressed how to improve pregnancy outcomes in women with multiple previous implantation failure, but the impact of a history of RIF on perinatal outcome has rarely been studied. Increasing evidences suggest that infants born after ART treatment are associated with a higher risk of low birth weight (LBW), preterm birth (PTB), small for gestational age (SGA), and also have an increased incidence of gestational diabetes, hypertensive disorders, and placenta previa, in both singleton and multiple pregnancies as compared with spontaneous conception (7–9). It is of great importance to investigate whether women with a history of multiple *in vitro* fertilization-embryo transfer (IVF-ET) failure are at higher risk for adverse neonatal and obstetric outcomes once they finally achieve a successful pregnancy. In this single-center study, we aimed to explore perinatal outcomes of singleton live births in patients with RIF in comparison to those who underwent a first FET cycle, in order to provide closer surveillance during pregnancy.

## Material and Methods

### Study Design and Population

This retrospective cohort study analyzed the obstetric and neonatal follow-up of singleton pregnancies following FET cycles at the Reproductive Center of the Third Affiliated Hospital of Zhengzhou University from January 2017 to October 2020. Eligible patients were women with a history of RIF who had previously undergone at least three embryo transfer cycles and transferred no less than four good-quality cleavage embryos or three blastocysts without obtaining a pregnancy and those who accepted a first IVF/intracytoplasmic sperm injection (ICSI) treatment. All female participants were under 40 years of age. The exclusion criteria were: sperm/oocyte donation cycles, pre-implantation genetic testing (PGT) cycles, and patients with diabetes, chronic hypertension, thrombophilia, thyroid and autoimmune abnormalities. Beyond these, patients with chromosomal abnormalities, congenital uterine malformations, endometrial polyps, submucosal uterine fibroids, endometriosis, adenomyosis or hydrosalpinx, and whose endometrial thickness on the transfer day was <7 mm were also excluded. In view of the retrospective nature of our study, the collection and processing of the data of patients were approved by the Ethics Review Committee of the Third Affiliated Hospital of Zhengzhou University (protocol number 2021-052-01).

### IVF/ICSI Protocols

The GnRH agonist protocol and GnRH-antagonist protocol were conventional controlled ovarian stimulation (COS) protocols. For the GnRH-agonist protocol, a standard dose of triptorelin (Diphereline, Ipsen Pharma, France) was administered in the early follicular phase or luteal phase of the previous cycle for pituitary downregulation. Downregulation [LH <5 IU/L, serum estradiol <50 pg/ml] was confirmed after 15–30 d. Exogenous Gn (Gonal-F, Merck Serono, Switzerland) was administered at doses ranging between 112.5 and 300 IU/d, generally in accordance with age, body mass index (BMI), basal FSH, size and number of follicles, and estradiol levels until the follicles reached maturity. The GnRH antagonist protocol is well described in our recent study (10). When at least 40% of follicles measured >18 mm, human chorionic gonadotrophin (hCG, Merck Serono, Switzerland) or GnRH agonist (Dophereline, Ipsen Pharma Biotech, France) was administered to trigger oocyte maturation. Oocyte retrieval was conducted under transvaginal ultrasound guidance 36–38 h after trigger. IVF or ICSI were then performed, according to the sperm quality, approximately 4 h after oocyte retrieval.

### Laboratory Protocols

All embryos were cultured at 37°C in 5% O_2_ and 6% CO_2_ G-1 Plus medium. Fertilization was observed 16–18 h after insemination. On Day 3 of embryo culture, embryo morphological evaluation was performed according to Cummins’ criteria based on the percentage of fragmentation and the size and number of blastomeres (11). Good quality cleavage embryos were assessed as at least grade II embryos. In our center, 1–2 cleavage embryos of good quality are usually selected for fresh embryo transfer or cryopreservation, while the remaining embryos are transferred to blastocyst G-2 Plus medium for subsequent culture and then cryopreserved. Blastocyst quality was evaluated on Day 5 or Day 6 according to Gardner and Schoolcraft’s criteria (12) based on the degree of blastocoel expansion, and morphology of the inner cell mass (ICM) and trophectoderm (TE). The cleavage embryos and blastocysts were scored by the same experienced embryologist, thus eliminating intra-embryologist bias when evaluating embryos. The frozen-thawed procedure is described in detail elsewhere (13).

### Endometrial Preparation

In subsequent FET cycles, patients usually received a hormone replacement therapy (HRT) cycle, a natural cycle, GnRHa-HRT cycle or stimulated cycle protocols for endometrial preparation before embryo transfer. In general, natural cycles were used for women with regular menstrual cycles who had the growth of their dominant follicle monitored by transvaginal ultrasonography from the 10th day of the menstrual cycle. When the diameter of the dominant follicle reached 14 mm, patients underwent a daily ovulation urine test for LH surge detection. Ovulation occurred spontaneously or was triggered by human chorionic gonadotropin (5,000–10,000 IU; Livzon) when the urine LH surge was detected. Oral dydrogesterone (Duphaston; Solvay Pharmaceuticals BV) 10 mg three times daily was started on the day after ovulation for luteal phase support. In stimulated cycles, women received letrozole (2.5–5.0 mg/d started on the 3rd–5th d of the menstrual cycle) for 5 d, with or without human menopausal gonadotropin (75–150 IU; Lizhu, Pharmaceutical) injection according to the growth of the dominant follicle; other procedures were the same as for the natural cycle.

Meanwhile, the HRT cycle was used for women with irregular menstrual cycles who received oral estradiol valerate (ValieraVR; Laboratories Recalcine) 4–8 mg/d for 12 d starting from the 2nd or 3rd d of menstruation and the estradiol dose could be increased to a maximum of 12 mg/d depending on the thickness of the endometrium. Oral dydrogesterone (Duphaston; Solvay Pharmaceuticals BV) 10 mg twice daily plus progesterone sustained-release vaginal gel (Xenotong, Merck Sherano, Switzerland) 90 mg daily was started when endometrial thickness reached ≥7 mm. For the GnRHa-HRT cycles, patients received downregulation therapy (Dophereline, Ipsen Pharma Biotech, France) on the 2nd to 3rd day of the menstrual cycle and then the protocol was the same as for HRT cycles when pituitary downregulation reached a satisfactory standard. Only 1–2 frozen-thawed embryos were transferred on the 6th d of progesterone administration. Luteal support was continued to 7 weeks of gestation if a pregnancy occurred.

### Outcome Measures

Live birth was defined as birth exhibiting any signs of life after 28 weeks of gestation. In our study, the primary neonatal parameters included gestational age, preterm birth (PTB, <37 weeks), very preterm birth (VPTB, <32 weeks), birth weight, low birth weight (LBW, <2500 g), very low birth weight (VLBW, 1,500 g), small for gestational age (SGA, birthweight is below the 10th percentile of the average body weight at the same gestational age), large for gestational age (LGA, birthweight is above the 90th percentile of the average body weight at the same gestational age), newborn sex, and congenital malformations.

In addition, we also analyzed the obstetric complications among singleton live births, including gestational hypertensive disorders, gestational diabetes mellitus (GDM), placenta previa, and premature rupture of the membranes (PROM). Gestational hypertension was defined as at least two blood pressure measurements ≥140/90 mmHg after 20 weeks of gestation, 6 h apart.

### Statistical Analysis

All data were analyzed by SPSS 25.0 statistical software. First, we matched the sample by 1:3 propensity score matching (PSM) between the RIF group and control group. The one-sample Kolmogorov–Smirnov (K–S) test was used to check for normality. Normally distributed continuous variables were expressed as mean ± standard deviation and compared between two groups by Student’s *t*-test. Categorical data were presented as frequencies (percentages), and were compared using Pearson chi-square or Fisher’s exact test as appropriate.

Univariable and multivariable logistic regression analyses were used to further explore the association between RIF and adverse obstetric and neonatal outcomes. The results are represented as odds ratio (OR), adjusted odds ratio (aOR) and 95% confidence interval (CI). In the subgroup analysis, multivariate regression analysis was performed for possible related variables affecting the incidence of PROM in RIF patients. *P <*0.05 was considered statistically significant.

## Results

A total of 1,100 singleton deliveries from FET cycles met the inclusion criteria, with 276 (25.09%) in the RIF group and 824 (74.91%) in the control group. The baseline demographics and main cycle characteristics for women with singleton live deliveries are described in **Table 1**. There were no significant differences in maternal age, maternal BMI, cause of infertility, basal FSH, and endometrial thickness on the transfer day between the two groups (*P >*0.05). Years of infertility (3.32 ± 2.60 vs. 3.90 ± 2.85; *P* = 0.003) and AMH level (4.31 ± 2.01 vs. 4.73 ± 2.95; *P* = 0.028) were lower in the RIF group than in the control group. The proportion of secondary infertility (66.30% vs. 50.36%; *P <*0.001), transfer of two embryos (61.96% vs. 21.37%; *P <*0.001) and the number of intrauterine procedures (5.75 ± 1.50 vs. 1.57 ± 0.92; *P <*0.001) were significantly higher in the RIF group compared with the control group. The RIF group showed a preferred transfer of cleavage embryos. Additionally, the FET endometrial preparation protocol was also significantly different between the groups (*P <*0.05).

**Table 1:** Demographic characteristics of women with a singleton birth following FET in the RIF group and control group.

Characteristic	RIF group	Control group	*P-*value
	(n = 276)	(n = 824)	
Maternal age (y)	31.92 ± 3.73	31.46 ± 3.63	0.073
Maternal BMI (kg/m^2^)	23.43 ± 3.03	23.75 ± 3.26	0.143
Years of infertility (y)	3.32 ± 2.60	3.90 ± 2.85	0.003
Infertility type, n (%)			<0.001^a^
Primary infertility	93 (33.70)	409 (49.64)	
Secondary infertility	183 (66.30)	415 (50.36)	
Cause of infertility, n (%)			0.528^a^
Tubal factor	97 (35.14)	298 (36.17)	
Ovulatory factor	46 (16.67)	154 (18.69)	
Male factor	49 (17.75)	130 (15.78)	
Combined factor	61 (22.10)	174 (21.12)	
Unexplained	23 (8.33)	68 (8.25)	
Basal FSH (IU/L)	6.78 ± 2.69	6.60 ± 2.46	0.327
AMH (ng/ml)	4.31 ± 2.01	4.73 ± 2.95	0.028
Endometrial preparation protocol, n (%) (%)			<0.001^a^
HRT cycles	130 (47.10)	359 (43.57)	
Natural cycles	79 (28.62)	330 (40.05)	
GnRHa-HRT cycles	41 (14.86)	25 (3.03)	
Stimulated cycles	26 (9.42)	110 (13.35)	
Endometrial thickness on transfer day(mm)	9.38 ± 1.61	9.48 ± 1.68	0.391
Number of transferred embryos, n (%)			<0.001^a^
1	105 (38.04)	582 (70.63)	
2	171 (61.96)	242 (29.37)	
Type of transferred embryos, n (%)			0.020^a^
Cleavage embryos	100 (36.23)	237 (28.76)	
Blastocyst embryos	176 (63.77)	587 (71.24)	
Number of intrauterine procedures (n)	5.75 ± 1.50	1.57 ± 0.92	<0.001

Values are presented as mean ± standard deviation or n (%). RIF, recurrent implantation failure; BMI, body mass index; FSH, follicle stimulating hormone; AMH, anti-Mullerian hormone; FET, frozen-thawed embryo transfer; HRT, hormones replacement therapy.

a Pearson’s chi-square test.

Neonatal outcomes and obstetric complications for singleton births are shown in **Table 2**. The mean gestational age of delivery was lower in the RIF group than in the control group (271.64 ± 13.72 d vs. 273.79 ± 10.75 d; *P* = 0.008). The rates of LBW were higher among infants in the RIF group than those of infants in the control group (6.16% vs. 3.52%). The risk of being born PTB (10.87% vs. 6.31%) and VPTB (1.81% vs. 0.49%) were significantly increased in the RIF group compared with those from the control group. Similarly, the RIF group was associated with a significant incidence of PROM (6.88% vs. 3.64%, *P* = 0.024). However, no significant differences were observed in terms of the rates of VLBW, SGA, LGA, male gender, congenital malformation, hypertensive disorders, GDM, and placenta previa. After discovering that the RIF group showed a trend toward an increased risk of adverse obstetric and neonatal outcomes, we then performed multivariate logistic regression analysis to further investigate the association between RIF and these perinatal outcomes, respectively (**Table 2**). After adjusting for the years of infertility, infertility type, AMH, FET endometrial preparation protocol, number of transferred embryos, type of transferred embryos, and the number of intrauterine procedures, the frequencies of LBW were still higher in the RIF group compared to the control group (aOR 2.027; 95% CI: 1.025–4.009; *P* = 0.042). Likewise, higher risks of PTB were also observed in RIF patients (aOR 1.785; 95% CI, 1.050–3.036; *P* = 0.032). Furthermore, compared with the control group, women in the RIF group were still associated with a significant increased risk of PROM (aOR 2.259; 95% CI: 1.142–4.467; *P* = 0.019).

**Table 2:** Neonatal outcomes and obstetric complications for singleton births in the RIF group versus control group.

Outcomes	RIF (n = 276)	Control (n = 824)	OR (95%CI)	*P* value	aOR (95% CI)	*P* value
Gestational age (d)	271.64 ± 13.72	273.79 ± 10.75		0.008		
PTB	30 (10.87)	52 (6.31)	1.811 (1.130–2.902)	0.014	1.785 (1.050–3.036)	0.032
VPTB	5 (1.81)	4 (0.49)	3.782 (1.008–14.186)	0.049	3.521 (0.791–15.675)	0.099
Birth weight (g)	3,393.45 ± 584.81	3,421.49 ± 503.86		0.443		
LBW	17 (6.16)	29 (3.52)	2.182 (1.193–3.990)	0.011	2.027 (1.025–4.009)	0.042
VLBW	2 (0.72)	1 (0.12)	6.007 (0.543–66.508)	0.144	4.015 (0.290–55.609)	0.300
SGA	10 (3.62)	28 (3.40).	1.069 (0.512–2.229)	0.859	1.190 (0.521–2.717)	0.680
LGA	54 (19.57)	150 (18.20)	1.093 (0.773–1.545)	0.615	1.192 (0.809–1.758)	0.375
Male gender	158 (57.25)	455 (55.22)	0.921 (0.669–1.213)	0.557	1.046 (0.769–1.422)	0.775
Congenital malformations	4 (1.45)	10 (1.21)	1.197 (1.130–2.902)	0.763	0.415 (0.100–1.718)	0.225
Hypertensive disorders	23 (8.33)	43 (5.22)	1.651 (0.976–2.793)	0.062	1.430 (0.788–2.595)	0.240
GDM	22 (7.97)	66 (8.01)	0.995 (0.602–1.645)	0.984	0.895 (0.508–1.579)	0.703
Placenta previa	2 (0.72)	10 (1.21)	0.594 (0.129–2.728)	0.503	0.576 (0.112–2.967)	0.510
PROM	19 (6.88)	30 (3.64)	1.957 (1.083–3.536)	0.026	2.259 (1.142–4.467)	0.019

OR, odds ratio; aOR, adjusted odds ratios; CI, confidence interval; PTB, preterm birth; VPTB, very preterm birth; LBW, low birth weight; VLBW, very low birth weight; SGA, small for gestational age; LGA, large for gestational age; GDM, gestational diabetes mellitus; PROM, premature rupture of the membranes. Adjusted for years of infertility, infertility type, AMH, FET endometrial preparation protocol, number of transferred embryos, type of transferred embryos and number of intrauterine procedures.

In subgroup analysis, patients with PROM in the RIF group experienced more intrauterine procedures than those without PROM (6.53 ± 0.96 vs. 5.64 ± 1.00; *P* = 0.012). After adjusting for maternal age, maternal BMI, type of infertility, basal FSH, AMH, number of transferred embryos, type of transferred embryos, and number of intrauterine procedures, multivariable logistic analysis showed that a multiple number of intrauterine procedures was an independent risk factor for PROM in RIF patients (aOR 1.537; 95% CI: 1.105–2.137; *P* = 0.014), as show in **Table 3**.

**Table 3:** Adjusted odds ratios of premature rupture of the membranes by multivariate analysis of predictor variables in RIF patients.

Predictor variable	aOR (95% CI)	*P*-value
Maternal age	0.987 (0.857–1.136)	0.853
Maternal BMI	1.104 (0.951–1.281)	0.193
Type of infertility		
Primary infertility	1	
Secondary infertility	0.599 (0.201–1.781)	0.356
Basal FSH	1.020 (0.846–1.230)	0.836
AMH	1.229 (1.005–1.504)	0.051
Number of transferred embryos		
1	2.044 (0.557–7.498)	0.281
2	1	
Type of transferred embryos		
Cleavage embryos	1.680 (0.459–6.153)	0.433
Blastocyst embryos	1	
Number of intrauterine procedures	1.537 (1.105–2.137)	0.014

aOR, adjusted odds ratios; CI, confidence interval; BMI, body mass index; FSH, follicle stimulating hormone; AMH, anti-Mullerian hormone.

## Discussion

The results of this retrospective cohort analysis indicated that patients with a history of RIF are associated with adverse perinatal outcomes in singleton live births following FET cycles. We found that 6.16, 10.87, and 6.88% of RIF patients had LBW, PTB, and PROM, respectively. The risk of the above three adverse outcomes increased approximately two-fold in women with RIF compared with those from the control group. In addition, subgroup analyses showed that a multiple number of intrauterine procedures was a risk factor for PROM.

One reason for this study was that women with RIF have been shown to have an elevated risk of adverse perinatal outcomes. Earlier studies found that patients with a history of RIF had a significantly increased risk of placental abruption among singleton pregnancies (14), but our study did not find this difference. To our knowledge, although the sample size of our study was limited, this is the first report to analyze the relationship between RIF and neonatal outcomes and obstetric complications in singleton live births only after FET cycles.

Our study showed singleton newborns conceived by women with a history of RIF are more likely to exhibit LBW. Preterm birth and intrauterine fetal growth restriction have long been known to be major factors affecting LBW. Premature infants are limited by the time of birth, that is, the duration of pregnancy is short, and the fetus is underdeveloped in the mother’s uterus, resulting in a significantly higher incidence of LBW than full-term infants. The research of Christine et al. and Victora et al. shows that premature delivery can significantly increase the incidence of LBW (15, 16). In our cohort, we found that 18 of 19 LBW infants in the RIF group were caused by PTB, which further showed that PTB is an important factor leading to LBW.

Additionally, the current study also showed that RIF was associated with statistically significant increased risks of PTB and PROM. Preterm delivery reflects the growth of the fetus and is strongly associated with neonatal mortality, which may have a great impact on a child’s health in the long run. It is not difficult to understand that PROM itself can cause premature delivery. Emma et al. postulated that defective implantation causes pregnancy loss, while partially impaired implantation may lead to placenta-associated complications (17). Furthermore, with the intensive study of the mechanisms involved in embryo-endometrial cross-talk and embryo implantation, most patients with RIF also exhibit an increased blood flow resistance of the spiral artery, which will reduce blood supply to the placenta and lead to placental ischemia and hypoxia, finally resulting in adverse pregnancy outcomes (18–20).

To our surprise, we also noted that multiple intrauterine procedures were an independent risk factor for PROM. When it comes to treatment of RIF patients, the most commonly proposed, used, and studied interventions to overcome RIF are preconception therapies, and experimental intrauterine therapies including endometrial scratching, endometrial receptivity analysis, hysteroscopy, and intrauterine G-CSF administration (21–24). The types of therapy aim to correct disorders of endometrial receptivity, which in turn improve reproductive outcomes in RIF patients. The pathophysiologic mechanisms include an increase in the release of growth factors, interleukins, cytokines, and dendritic cell cytokines, and the induction of endometrial decidualization (25). However, results from these clinical trials are currently conflicting in women with RIF undergoing ART treatment and the procedure-associated complications have not been assessed (26). It is well-known that the intrauterine environment plays an important role in maternal and fetal development during pregnancy (27). IVF-ET procedures involve embryo catheter transfer and experimental intrauterine therapies through the cervix, which may result in vaginal–cervical dysbacteriosis, while the ascending infection of vaginal microorganisms into the uterus may trigger an inflammatory response, ultimately leading to PROM (28). We therefore hypothesize that intrauterine procedures alter the colonization of the intrauterine microbiota in RIF patients. In order to support the hypothesis, we will further investigate the inflammatory sequelae of intrauterine operations and the effects of inflammation on PROM.

The strength of our study is that it was performed at a single reproductive center, and we considered the risk factors associated with the incidence of PROM. Secondly, we excluded relevant variables known to affect perinatal outcomes. Lastly, we limited the analysis to only patients with a singleton live birth delivered from FET cycles. There were, however, some limitations in our study. First of all, a retrospective cohort study has inherent limitations. Secondly, our database lacked data on parental lifestyle habits, such as smoking and drinking, which have been shown to increase the risk of adverse pregnancy outcomes. Above all, the relevant biological mechanisms of intrauterine procedures on PROM need to be further explored.

### Conclusions

In summary, our study demonstrated that women with a history of RIF are associated with an increased risk of LBW, PTB, and PROM in singleton live births conceived after FET cycles. Most importantly, multiple intrauterine procedures are a risk factor for PROM. Therefore, we suggest that RIF patients should minimize unnecessary intrauterine procedures. In addition, we should strengthen antenatal care and prenatal surveillance during pregnancy to reduce the risk of adverse perinatal outcomes.

## Data Availability Statement

The raw data supporting the conclusions of this article will be made available by the authors, without undue reservation.

## Ethics Statement

The studies were reviewed and approved by the Ethics Review Committee of the Third Affiliated Hospital of Zhengzhou University. Written informed consent for participation was not required for this study in accordance with the national legislation and the institutional requirements.

## Author Contributions

HL and YG proposed the design ideas. NL, BR, and YD acquired and analyzed the data. NL, KW, and YZ prepared all tables and figures. NL wrote the manuscript. HL and JL revised the manuscript. All authors contributed to the article and approved the submitted version.

## Funding

This study was supported by grant 2018020198 from the Henan Medical Science and Technology Research Project, China.

## Conflict of Interest

The authors declare that the research was conducted in the absence of any commercial or financial relationships that could be construed as a potential conflict of interest.

## Publisher’s Note

All claims expressed in this article are solely those of the authors and do not necessarily represent those of their affiliated organizations, or those of the publisher, the editors and the reviewers. Any product that may be evaluated in this article, or claim that may be made by its manufacturer, is not guaranteed or endorsed by the publisher.

## References

[1] Margalioth EJ Ben-Chetrit A Gal M Eldar-Geva T. Investigation and Treatment of Repeated Implantation Failure Following IVF-ET. Hum Reprod (Oxf Engl) (2006) 21(12):3036–43. doi: 10.1093/humrep/del305 16905766

[2] Coughlan C Ledger W Wang Q Liu F Demirol A Gurgan T. Recurrent Implantation Failure: Definition and Management. Reprod BioMed Online (2014) 28(1):14–38. doi: 10.1016/j.rbmo.2013.08.011 24269084

[3] Alex S Neri L. Assessment and Treatment of Repeated Implantation Failure (RIF). J Assist Reprod Genet (2012) 29(11):1227–39. doi: 10.1007/s10815-012-9861-4 PMC3510376 22976427

[4] Sar-Shalom Nahshon C Sagi-Dain L Wiener-Megnazi Z Dirnfeld M. The Impact of Intentional Endometrial Injury on Reproductive Outcomes: A Systematic Review and Meta-Analysis. Hum Reprod Update (2019) 25(1):95–113. doi: 10.1093/humupd/dmy034 30388238

[5] Neelam P Tarek G Luciano GN. Endometrial Injury to Overcome Recurrent Embryo Implantation Failure: A Systematic Review and Meta-Analysis. Reprod BioMed Online (2012) 25(6):561–71. doi: 10.1016/j.rbmo.2012.08.005 23063812

[6] Claudia S Catherine K Cordula F Kaino H Osama M Manfred S. Granulocyte-Colony Stimulating Factor as Treatment Option in Patients With Recurrent Miscarriage. Arch Immunol Ther Exp (2013) 61(2):159–64. doi: 10.1007/s00005-012-0212-z 23344173

[7] Burton GJ Jauniaux E. Pregnancy, Birth, and Infant Outcomes by Maternal Fertility Status: The Massachusetts Outcomes Study of Assisted Reproductive Technology. Am J Obstet Gynecol (2017) 217(3):327.e1–.e14. doi: 10.1016/j.ajog.2017.04.025 PMC5581226 28400311

[8] Qin J Liu X Sheng X Wang H Gao S. Assisted Reproductive Technology and the Risk of Pregnancy-Related Complications and Adverse Pregnancy Outcomes in Singleton Pregnancies: A Meta-Analysis of Cohort Studies. Fertil Steril (2016) 105(1):73–85.e1-6. doi: 10.1016/j.fertnstert.2015.09.007 26453266

[9] Henningsen AK Pinborg A Lidegaard O Vestergaard C Forman JL Andersen AN. Perinatal Outcome of Singleton Siblings Born After Assisted Reproductive Technology and Spontaneous Conception: Danish National Sibling-Cohort Study. Fertil Steril (2011) 95(3):959–63. doi: 10.1016/j.fertnstert.2010.07.1075 20813359

[10] Lou H Li N Guan Y Zhang Y Hao D Cui S. Association Between Morphologic Grading and Implantation Rate of Euploid Blastocyst. J Ovarian Res (2021) 14(1):18. doi: 10.1186/s13048-021-00770-8 33485390 PMC7827997

[11] Cummins J Breen T Harrison K Shaw J Wilson L Hennessey J. A Formula for Scoring Human Embryo Growth Rates in *In Vitro* Fertilization: Its Value in Predicting Pregnancy and in Comparison With Visual Estimates of Embryo Quality. J In Vitro Fert Embryo Transf (1986) 3(5):284–95. doi: 10.1007/BF01133388 3783014

[12] Gardner DK Lane M Stevens J Schlenker T Schoolcraft WB. Blastocyst Score Affects Implantation and Pregnancy Outcome: Towards a Single Blastocyst Transfer. Fertil Steril (2000) 73(6):1155–8. doi: 10.1016/s0015-0282(00)00518-5 10856474

[13] Lou H Li N Zhang X Sun L Wang X Hao D. Does the Sex Ratio of Singleton Births After Frozen Single Blastocyst Transfer Differ in Relation to Blastocyst Development? Reprod Biol Endocrinol (2020) 18(1):72. doi: 10.1186/s12958-020-00623-x 32669110 PMC7362517

[14] Chin TH Hsu YC Soong YK Lee CL Wang HS Huang HY. Obstetric and Perinatal Outcomes of Pregnancy in Patients With Repeated Implantation Failure. Taiwan J Obstet Gynecol (2019) 58(4):487–91. doi: 10.1016/j.tjog.2019.05.010 31307738

[15] Victora C Aquino E do Carmo Leal M Monteiro C Barros F Szwarcwald C. Maternal and Child Health in Brazil: Progress and Challenges. Lancet (London England) (2011) 377(9780):1863–76. doi: 10.1016/S0140-6736(11)60138-4 21561656

[16] Odell C Kotelchuck M Chetty V Fowler J Stubblefield P Orejuela M. Maternal Hypertension as a Risk Factor for Low Birth Weight Infants: Comparison of Haitian and African-American Women. Matern Child Health J (2006) 10(1):39–46. doi: 10.1007/s10995-005-0026-2 16397832

[17] BG J Eric J. Placental Oxidative Stress: From Miscarriage to Preeclampsia. J Soc Gynecol Investig (2004) 11(6):341–52. doi: 10.1016/j.jsgi.2004.03.003 15350246

[18] Kitaya K Matsubayashi H Takaya Y Nishiyama R Yamaguchi K Takeuchi T. Live Birth Rate Following Oral Antibiotic Treatment for Chronic Endometritis in Infertile Women With Repeated Implantation Failure. Am J Reprod Immunol (2017) 78(5):1–8. doi: 10.1111/aji.12719 28608596

[19] Ruiz-Alonso M Blesa D Díaz-Gimeno P Gómez E Fernández-Sánchez M Carranza F. The Endometrial Receptivity Array for Diagnosis and Personalized Embryo Transfer as a Treatment for Patients With Repeated Implantation Failure. Fertil Steril (2013) 100(3):818–24. doi: 10.1016/j.fertnstert.2013.05.004 23756099

[20] Kliman H Frankfurter D. Clinical Approach to Recurrent Implantation Failure: Evidence-Based Evaluation of the Endometrium. Fertil Steril (2019) 111(4):618–28. doi: 10.1016/j.fertnstert.2019.02.011 30929719

[21] Vitagliano A Andrisani A Alviggi C Vitale S Valenti G Sapia F. Endometrial Scratching for Infertile Women Undergoing a First Embryo Transfer: A Systematic Review and Meta-Analysis of Published and Unpublished Data From Randomized Controlled Trials. Fertil Steril (2019) 111(4):734–46.e2. doi: 10.1016/j.fertnstert.2018.12.008 30683590

[22] El-Toukhy T Campo R Khalaf Y Tabanelli C Gianaroli L Gordts S. Hysteroscopy in Recurrent *in-Vitro* Fertilisation Failure (TROPHY): A Multicentre, Randomised Controlled Trial. Lancet (2016) 387(10038):2614–21. doi: 10.1016/S0140-6736(16)00258-0 27132053

[23] Hashimoto T Koizumi M Doshida M Toya M Sagara E Oka N. Efficacy of the Endometrial Receptivity Array for Repeated Implantation Failure in Japan: A Retrospective, Two-Centers Study. Reprod Med Biol (2017) 16(3):290–6. doi: 10.1002/rmb2.12041 PMC5715887 29259480

[24] Davari-Tanha F Shahrokh Tehraninejad E Ghazi M Shahraki Z. The Role of G-CSF in Recurrent Implantation Failure: A Randomized Double Blind Placebo Control Trial. Int J Reprod BioMed (2016) 14(12):737–42. PMC5203688 28066833

[25] Nastri C Lensen S Gibreel A Raine-Fenning N Ferriani R Bhattacharya S. Endometrial Injury in Women Undergoing Assisted Reproductive Techniques. Cochrane Database Syst Rev (2015) 3:CD009517. doi: 10.1002/14651858.CD009517.pub4 25803542

[26] Shaulov T Sierra S Sylvestre C. Recurrent Implantation Failure in IVF: A Canadian Fertility and Andrology Society Clinical Practice Guideline. Reprod BioMed Online (2020) 41(5):819–33. doi: 10.1016/j.rbmo.2020.08.007 32962928

[27] Chen HJ Gur TL. Intrauterine Microbiota: Missing, or the Missing Link? Trends Neurosci (2019) 42(6):402–13. doi: 10.1016/j.tins.2019.03.008 PMC6604064 31053242

[28] Moore D Soules M Klein N Fujimoto V Agnew K Eschenbach D. Bacteria in the Transfer Catheter Tip Influence the Live-Birth Rate After *In Vitro* Fertilization. Fertil Steril (2000) 74(6):1118–24. doi: 10.1016/s0015-0282(00)01624-1 11119737

